# *Ethylene‐responsive factor 4* is associated with the desirable rind hardness trait conferring cracking resistance in fresh fruits of watermelon

**DOI:** 10.1111/pbi.13276

**Published:** 2019-11-06

**Authors:** Nanqiao Liao, Zhongyuan Hu, Yingying Li, Junfang Hao, Shuna Chen, Qin Xue, Yuyuan Ma, Kejia Zhang, Ahmed Mahmoud, Abid Ali, Guy Kateta Malangisha, Xiaolong Lyu, Jinghua Yang, Mingfang Zhang

**Affiliations:** ^1^ Laboratory of Germplasm Innovation and Molecular Breeding Institute of Vegetable Science Zhejiang University Hangzhou China; ^2^ Key laboratory of Horticultural Plant growth Development and Quality Improvement Ministry of Agriculture Hangzhou China

**Keywords:** Watermelon, fresh fruit, rind hardness, bulk segregant analysis, Genetic map, fine mapping, cracking resistance, *ClERF4*

## Abstract

Fruit rind plays a pivotal role in alleviating water loss and disease and particularly in cracking resistance as well as the transportability, storability and shelf‐life quality of the fruit. High susceptibility to cracking due to low rind hardness is largely responsible for severe annual yield losses of fresh fruits such as watermelon in the field and during the postharvest process. However, the candidate gene controlling the rind hardness phenotype remains unclear to date. Herein, we report, for the first time, an *ethylene‐responsive transcription factor 4* (*ClERF4*) associated with variation in rind hardness via a combinatory genetic map with bulk segregant analysis (BSA). Strikingly, our fine‐mapping approach revealed an InDel of 11 bp and a neighbouring SNP in the *ClERF4* gene on chromosome 10, conferring cracking resistance in F_2_ populations with variable rind hardness. Furthermore, the concomitant kompetitive/competitive allele‐specific PCR (KASP) genotyping data sets of 104 germplasm accessions strongly supported candidate *ClERF4* as a causative gene associated with fruit rind hardness variability. In conclusion, our results provide new insight into the underlying mechanism controlling rind hardness, a desirable trait in fresh fruit. Moreover, the findings will further enable the molecular improvement of fruit cracking resistance in watermelon via precisely targeting the causative gene relevant to rind hardness, *ClERF4*.

## Introduction

Fruit cracking, as an undesirable characteristic, is a serious genetic and physiological disorder in fresh fruits that severely reduces their market acceptability, and causes huge yield losses in fields and the following logistical chains annually. Fruit cracking is a complicated trait associated with hereditary and environmental factors (Capel *et al.*, 2017). Different genetic accessions possess large differences in cracking tolerance, indicating that genetic factors play a significant role in fruit cracking of sweet cherry (Correia *et al.*, 2018). Studies on fruit cracking are far fewer than those on other abiotic and biotic stresses, mainly due to the lack of effective experimental methods to induce cracking phenotypes (Capel *et al.*, 2017). Therefore, the exploration of relevant genetic populations and precise quantification of an effective index for fruit cracking are prerequisites to successfully map the candidate genes underlying the mechanism of fruit cracking.

The fruit rind plays an important role in fruit cracking, water loss, and disease and thus has a strong impact on fruit transportability, storability and shelf‐life quality. However, many rind‐associated traits, especially their mechanical properties, are too polygenic and sophisticated to be phenotyped and thus difficult to precisely target (Petit *et al.*, 2017). Studies on fruit rind‐associated traits, especially rind hardness, which confers cracking resistance, have been largely neglected over decades. Accordingly, information regarding the inheritance pattern of rind‐associated traits is elusive, and the candidate gene controlling rind hardness is lacking in fresh fruits such as watermelon. Nevertheless, the identification of causative genes controlling rind hardness and the development of corresponding molecular markers will enable further precision breeding using either CRISPR‐Cas9 or marker‐assisted selection toolkits.

Watermelon, as a popular fresh fruit, is an economically important cash crop grown globally. After the release of the watermelon genome (Guo *et al.*, 2013), watermelon has become an ideal model crop for research on traits such as fruit cracking, size, shape, rind colour, and flesh texture due to being an annual and thus having a shorter life cycle than other perennial fruit crops. With the advancement of next‐generation sequencing (NGS) technology, sequencing‐based gene mining strategies, such as bulk segregant analysis (BSA), genetic mapping and genome‐wide association study (GWAS), have been widely used as affordable, efficient and routine approaches to dissect crop traits in rice (Wang *et al.*, 2018b) tomato (Chapman *et al.*, 2012; Soyk *et al.*, 2017), cucumber(Li *et al.*, 2018b; Xu *et al.*, 2018), peanut(Luo *et al.*, 2018), chickpea(Deokar *et al.*, 2019),spinach(She *et al.*, 2018), apple(Jia *et al.*, 2018) and melon (Hu *et al.*, 2017). Recently, genes or QTLs related to sugar transporter (Ren *et al.*, 2018), dwarfism (Dong *et al.*, 2018) and lobed leaves (Wei *et al.*, 2017) have been reported in watermelon. However, the genetic dissection of rind hardness has not been reported and remains a knowledge gap in fresh fruits such as watermelon.

In this study, a Texture Analyzer TA.XT‐21 (Stable Micro Systems Ltd., Godalming, Surrey, UK) was effectively employed to phenotype the mechanical properties of watermelon rind. We discovered that rind hardness was positively correlated with fruit cracking characteristics and thus was shown to be an effective and reliable indicator to quantify the watermelon capacity for cracking resistance. We, for the first time, identified an *ethylene‐responsive transcription factor 4* (*ClERF4*) coupled with variations in rind hardness. Our fine‐mapping approach displayed an InDel of 11 bp and a neighbouring SNP in the *ClERF4* gene on chromosome 10 associated with variable rind hardness segregations in F_2_ populations. The resultant Kompetitive/competitive allele‐specific PCR (KASP) genotyping analysis of 104 germplasm accessions strongly supported candidate *ClERF4* as a causative gene responsible for rind hardness and thus conferring cracking resistance.

## Results

### Rind hardness in watermelon is quantitatively inherited and highly correlated with variations in fruit cracking resistance

**Figure 1:**
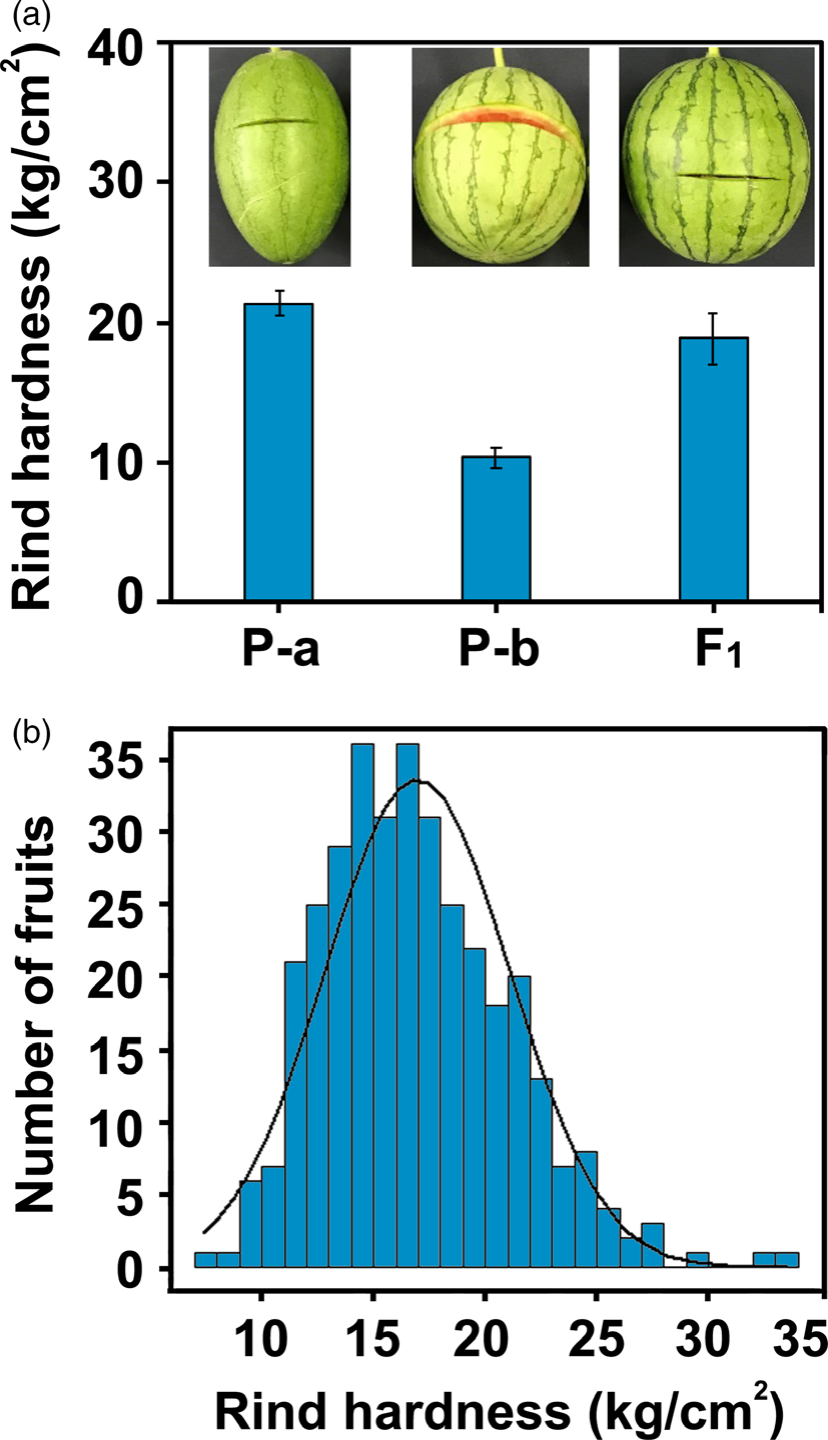
Rind hardness of cracking tolerant and cracking sensitive watermelon genotypes and their F_2_ population. (a) Rind hardness and cracking phenotypes of P‐a (high rind hardness, cracking tolerant), P‐b (low rind hardness, cracking sensitive) and their hybrid F_1_. (b) Frequency distribution of rind hardness among F_2_ individuals.

To precisely quantify the variability of mechanical properties for genetic analysis, a texture analyser was used to evaluate the mechanical properties of watermelon fruits. After treatment with an HDP/BS‐B knife probe (7.0 cm), the cracking length of P‐a was 7.5 cm, while the cracking length of P‐b was 28.2 cm, illustrating that P‐a was a cracking resistant line and that P‐b was a cracking susceptible line. In addition, the F_1_ of P‐a and P‐b showed a similar phenotype of cracking resistance to P‐a (Figure 1a). To find an effective index for fruit cracking in watermelon, the cracking work (CRW) and cracking time (CRT) from the texture characteristic curve were acquired under the HDP/BS‐B knife. A determination of cracking or not cracking (CRN) was obtained after the measurement. To confirm the relationship of rind hardness and cracking resistance capacity based on the CRW, CRT and CRN, the Pearson correlation coefficients (*r* ^2^) between rind hardness, CRW, CRT and CRN values were calculated using data from F_2_ populations. There was a significant positive correlation between rind hardness and the CRW, CRT and CRN (Table S1), which was also verified in 104 natural genetic accessions (Table S2), suggesting that rind hardness was a reliable indicator of the capacity for cracking resistance.

To further test the inheritance pattern of rind hardness, mature fruits were examined using a texture analyser to assess their rind hardness and cracking resistance capacity. Accordingly, the hardness values of P‐a and P‐b were 21.38 kg/cm^2^ and 10.33 kg/cm^2^, respectively (Figure 1a). A phenotypic analysis revealed that the rind hardness of the F_1_ population from the P‐a × P‐b crossing was 18.83 kg/cm^2^ on average, indicating an incomplete dominance of cracking resistance over cracking susceptibility. The variables of rind hardness in F_2_ populations displayed a largely normal distribution (Figure 1b), which was consistent with an inheritance pattern of major effect QTL traits.

### Construction of genetic map and identification of candidate QTL for rind hardness via QTL‐seq

**Figure 2:**
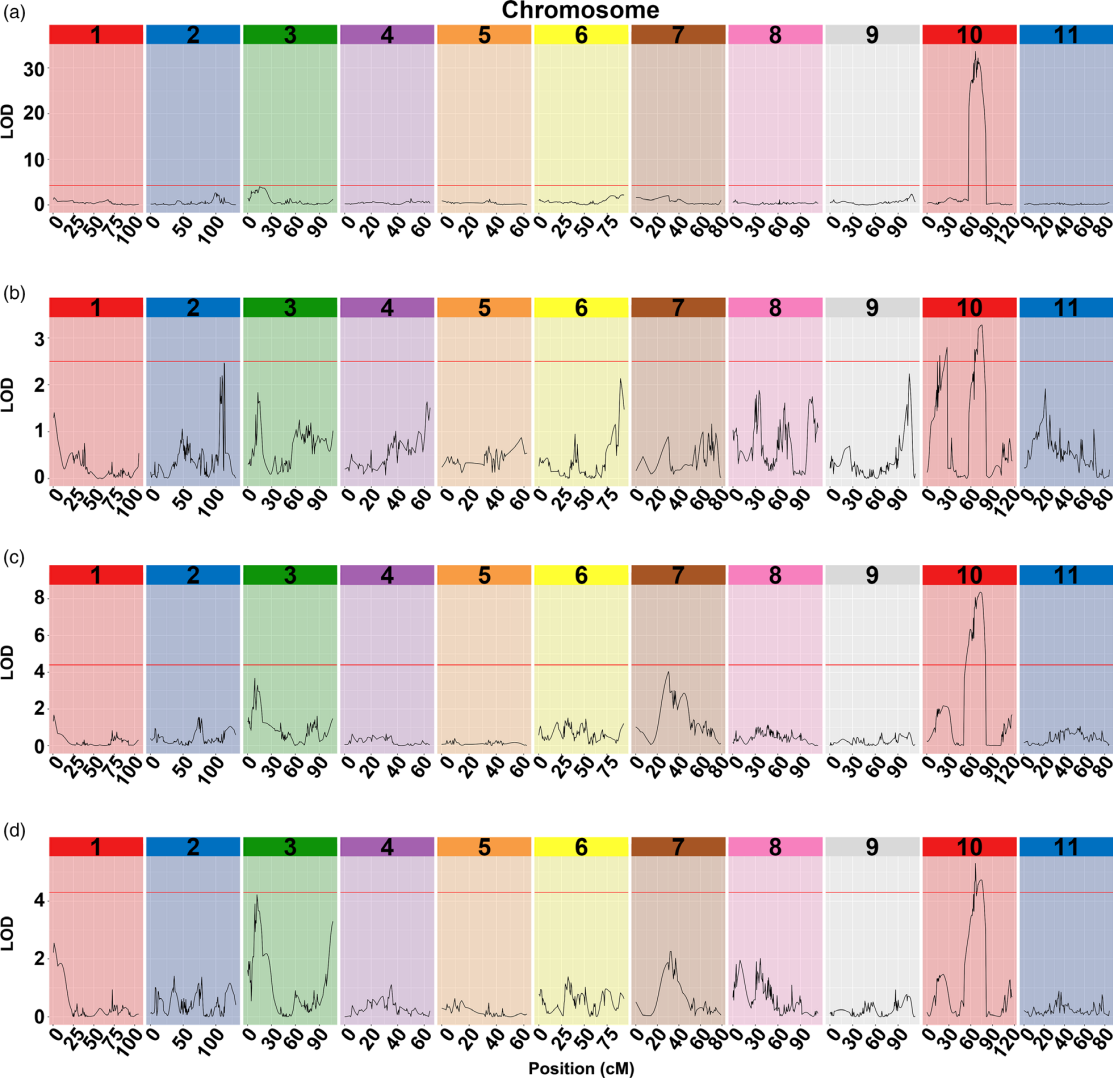
Colocation of rind hardness parameters and cracking‐related traits. (a) RH (rind hardness), (b) CRN (cracking or not), (c) CRW (cracking work), (d) CRT (cracking time).

To anchor the candidate QTLs responsible for rind hardness, linkage analysis was conducted to construct a genetic map using F_2_ populations. In total, 31.41 Gb clean reads were generated for P‐a and P‐b inbred lines (35 × genome coverage). Moreover, 493.80 Gb data were generated for 159 individuals from F_2_ populations (5 × genome coverage on average) with high quality (Q20 ≥ 93.56%, Q30 ≥ 84.56%; Table S3). The cosegregating SNPs were clustered in recombination bin makers, and a total of 5679 bin makers were used to construct the genetic map. The map consisted of 11 chromosomes and covered 1076.08 cM with an average distance of 0.19 cM (Fig. S1). QTLs of the cracking indicators CRT, CRW, CRN and rind hardness all pointed to colocalization on chromosome 10 (Fig. 2, Table S4).

**Figure 3:**
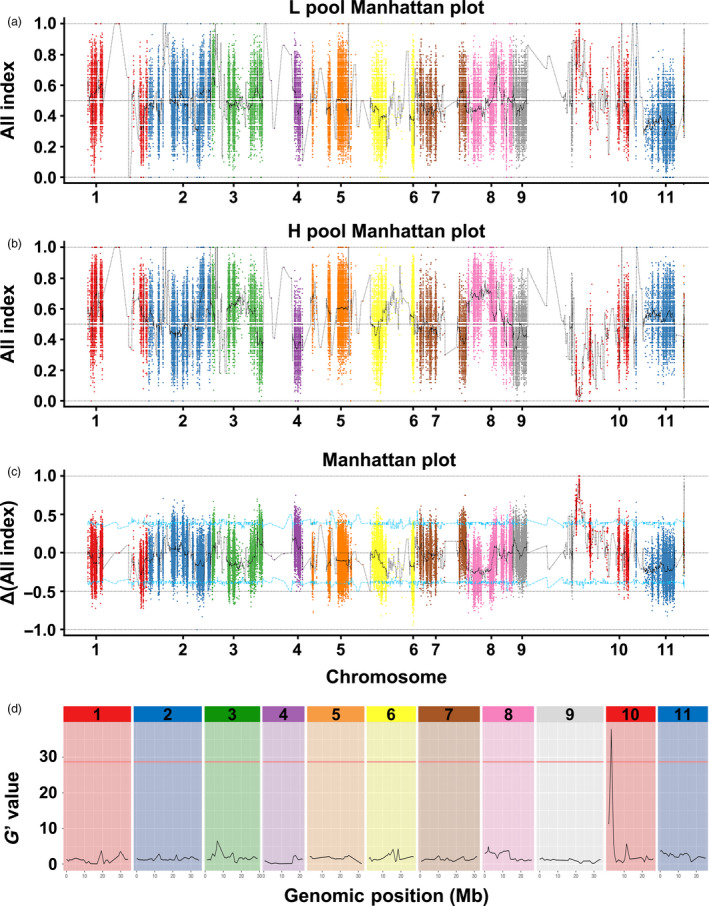
The results of BSA of watermelon rind hardness. (a) Graphs of the SNP index of the L‐pool, (b) graphs of the SNP index of the H‐pool and (c) the ΔSNP index values used for the association analysis. The *x*‐axis and *y*‐axis indicate the 11 watermelon chromosomes and the SNP index, respectively. The black line represents the fitted SNP index or ΔSNP index. The red, blue and green lines indicate the threshold for association with FFN at the 99%, 95% and 90% confidence interval, respectively. (d) Major quantitative trait loci for watermelon rind hardness identified by QTLseqr.

To further map the candidate gene for rind hardness, we first used the QTL‐seq approach to identify the candidate QTL. Genomic DNA of 20 low‐hardness F_2_ individuals and 20 high‐hardness individuals were evenly mixed as the L‐pool and H‐pool, respectively. Four pools (P‐a, P‐b, L‐pool and H‐pool) of DNA were sequenced by the Illumina HiSeq4000 platform and produced 50.653 G clean data (Table S5). Most of the data obtained were high quality, with Q20 ≥ 96.36% and Q30 ≥ 90.61%, and the G/C ratio was between 34.64% and 35.27% (Table S5). Ultimately, approximately 15.07, 13.86, 9.02 and 8.76 Gb clean reads were obtained from P‐a, P‐b, the L‐pool and the H‐pool, respectively (Table S5). The mapping rates of the four pools were 98.93%–99.19%, and the average depths of P‐a, P‐b, the L‐pool and the H‐pool were 34.52X, 31.42X, 21.93X and 22.60X, respectively. In total, 95,850 homozygous SNPs were called between two parents. For identifying SNPs, the SNP index of the L‐pool and H‐pool was calculated and plotted to the genome position (Fig. 3a, b) and then Δ (SNP index) was derived by subtracting the SNP index value of the H‐pool from the L‐pool (Fig. 3c). According to the null hypothesis, we chose peak regions above the threshold value as the candidate region harbouring major QTL for the target trait (Fig. 3c). With the 95% significance level and 99% significance level, we obtained 14 QTLs (Table S6) and 3 QTLs (Table S7) separately. We also analysed the data with QTLseqr and calculated the *G*′ value plotted to the genome position (Fig. 3d). With a 99% significance level, a genomic region (Cla97Chr10:2534127‐2801352) was found to have a *G*′ value above the threshold (Fig. 3d, Table S8). This region was also identified in the QTL‐seq results and was thus referred to as the target region harbouring a causal variant for rind hardness.

### Haplotype analysis and fine mapping of the candidate gene for rind hardness

**Figure 4:**
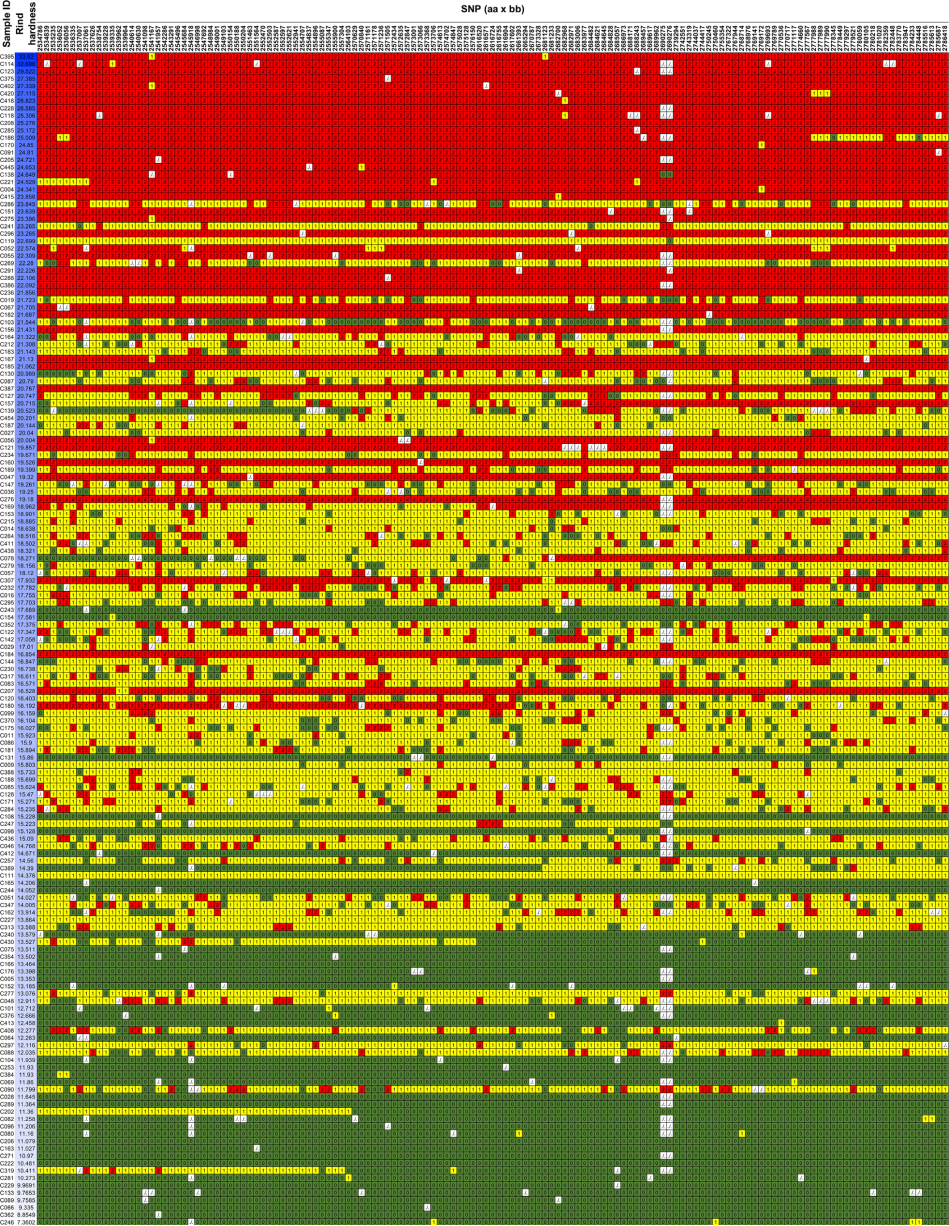
The visible data of SNPs in the target region. The haplotype of the 159 F_2_ individuals. Red indicates homozygous P‐a, green indicates homozygous P‐b, while yellow indicates heterozygous. The blue colour depth represents the rind hardness. The SNP data of individuals were arranged from top to bottom according to descending order of rind hardness values.

The SNPs in the target region were extracted from the VCF file document that was produced by the genetic map analysis. The markers were transformed into visible heatmap data as described in the materials and methods section. When the SNP data were arranged from top to bottom according to descending order of rind hardness, most homozygous chromosomes from P‐a were clustered in the top, and the homozygous chromosomes from P‐b were clustered in the bottom, while the heterozygous chromosomes from both P‐b and P‐a were clustered in the middle (Fig. 4). The data clearly supported the hypothesis that the target region was strongly associated with rind hardness.

**Figure 5:**
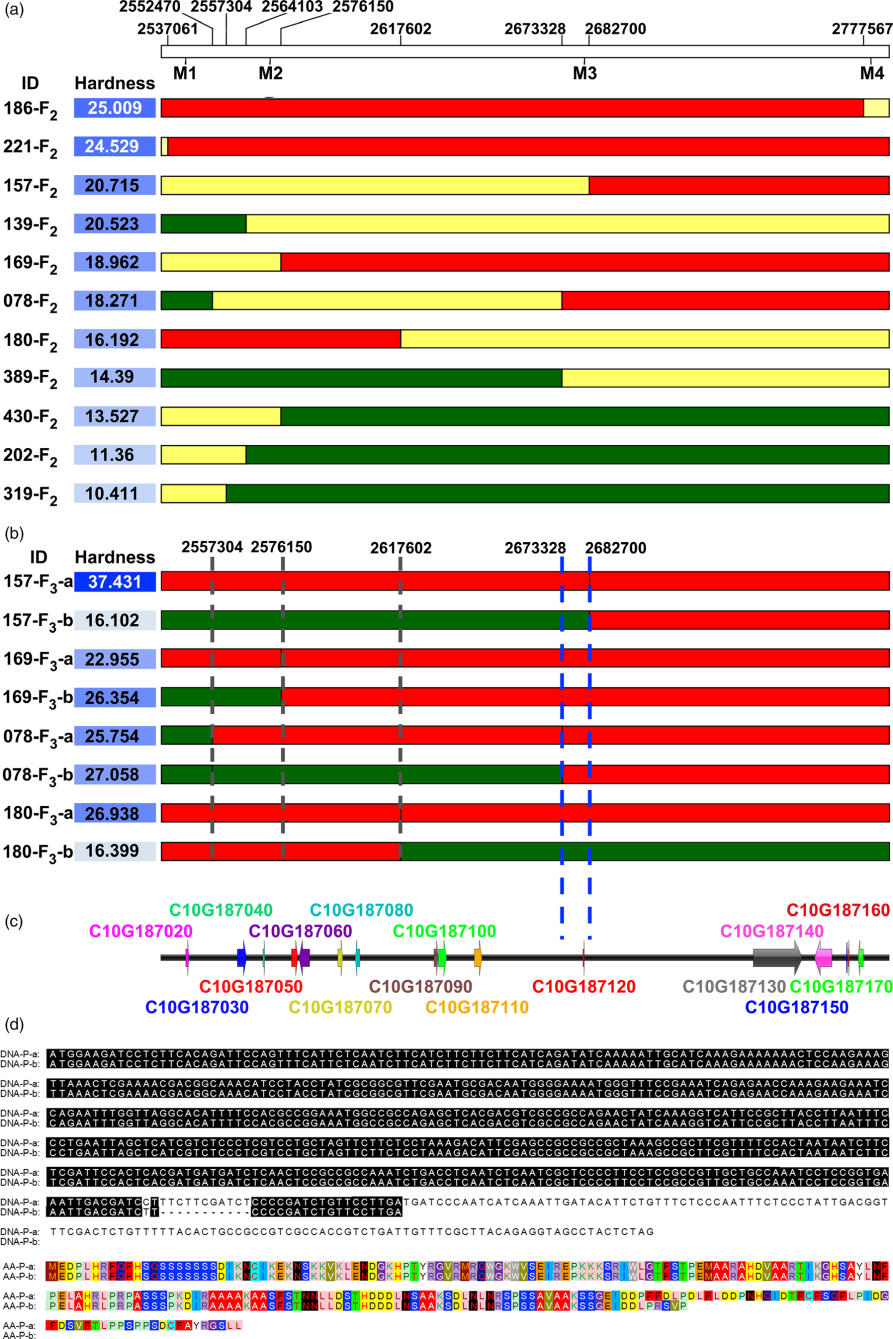
Fine mapping of the candidate gene. (a) Individuals with chromosome segment substitution on the target region, red indicates homozygous P‐a segment, green indicates homozygous P‐b segment, and yellow indicates heterozygous region. (b) The recombinants offspring of 157‐F_2_, 180‐F_2_ 169‐F_2_ and 078‐F_2_ were classified into two groups according to the origin of the recombinant segment. The average hardness of each group was measured from 3 fruits from 3 F_3_ individuals. (c) Candidate genes in the target region. (d) An InDel of 11 bp and SNP on the CDS of the candidate Cla97C10G187120 gene leading to two types of protein sequences.

Among the 159 individuals from the F_2_ population, there were 11 recombinants with chromosome segment substitution in the target region, and the exchange sites were visualized using the heat map (Fig. 5a). The offsprings of the recombinants (F_3_ self‐crossed from lines 157‐F_2_, 169‐F_2_, 078‐F_2_ and 180‐F_2_) were chosen for fine mapping, and four SNPs/InDels among the target region (site: Cla97Chr10:2543291; 2572434; 2681123; 2780105) were selected as KASP markers (Table S9), which were used in the selection of homozygote recombinants. The chromosome exchange site of recombinant 157‐F_2_ was approximately Cla97Chr10:2682700; the right segment of this line originated from the chromosome of P‐a, while the left region was heterozygous (Fig. 5a). The offspring of the 157‐F_2_ were classified into two groups, 157‐F_3_‐a and 157‐F_3_‐b, according to origin of the recombinant segment. Significant differences in rind hardness were observed between the two groups (Fig. 5b), suggesting the presence of a rind hardness‐related QTL in the recombinant segment. Similarly, the reorganization of the heterozygous segment in 180‐F_2_ resulted in significant hardness variations in 180‐F_3_‐a and 180‐F_3_‐b (Fig. 5b), indicating a left margin of the target region on Cla97Chr10:2617602. Meanwhile, the recombinant offspring of 078‐F_2_ exhibited no significant difference among the P‐a and P‐b types (Cla97Chr10:2557304–2682700; Fig. 5b), which strongly supported the region of interest (Cla97Chr10:2673328–2682700) as a causative region relevant to fruit rind hardness.

Indeed, among the target region, there was only one gene, Cla97C10G187120 (designed as *ClERF4*), which was predicted to encode an ethylene‐responsive transcription factor 4 (Fig. 5c). An 11‐bp InDel and a neighbouring SNP with a ‘C base’ in P‐a and a ‘T base’ in P‐b in the CDS of the gene were found to be translationally changed (Fig. 5d). Compared to P‐a, P‐b had an 11‐bp deletion as well as a neighbouring SNP, which resulted in a frame‐shift deletion and earlier termination and then caused two types of transcriptions, leading to two types of protein sequences (Fig. 5d).

### Allelic variations of *ClERF4* and validation of its role in rind hardness via KASP analysis

**Figure 6:**
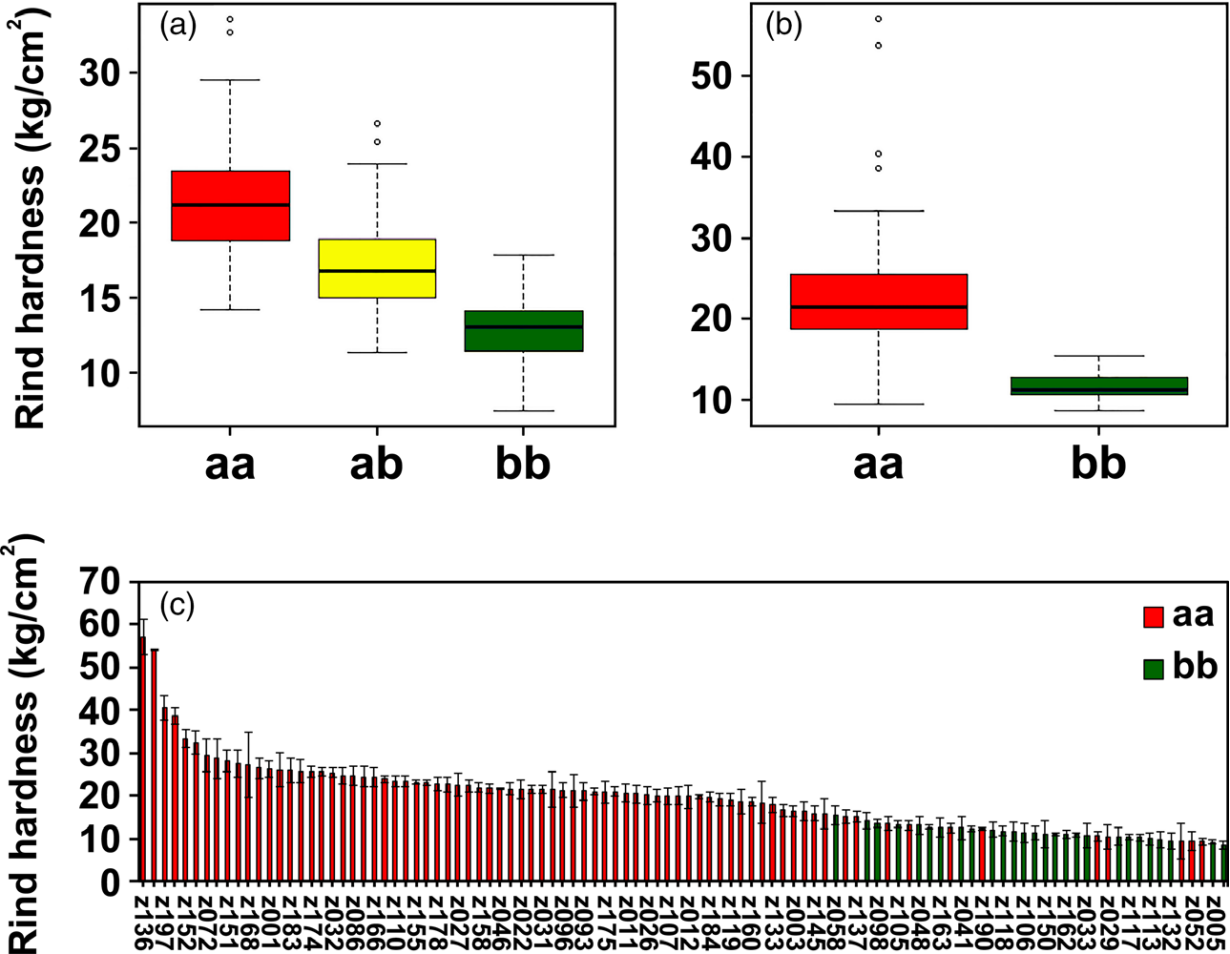
Association between the allelic distributions of *ClERF4* and rind hardness variation. (a) Association analysis between *ClERF4* genotypes (aa, ab and bb) and rind hardness in 349 F_2_ individuals from ‘P‐a’ × ‘P‐b’. (b) Association analysis between *ClERF4* genotypes (aa and bb) and rind hardness in 104 germplasm accessions. (c) The rind hardness and genotype of the 104 germplasm accessions.

To further study the allelic variations of the candidate *ClERF4* and its association with rind hardness, a KASP marker, M3, was developed for genotyping the candidate *ClERF4* (aa, ab and bb). Among 349 individuals from F_2_ populations of P‐a and P‐b, the rind hardness displayed a marked ascending pattern of aa > ab > bb (Fig. 6a, Table S10). Among the 104 germplasm accessions, the rind hardness of the aa genotype was significantly higher than that of bb (Fig. 6b, c, Table S11). To date, there have been two sequenced reference genomes for the watermelon accessions of ‘Charleston Gray’ and ‘97103’. In the present study, we discovered that the American dessert watermelon ‘Charleston Gray’ (Wu *et al.*, 2019), which has a thick and tough rind, showed the aa genotype in the *ClERF4* locus. The Chinese elite line ‘97103’ (Guo *et al.*, 2013) showed the bb type in the *ClERF4* locus. These data jointly support the hypothesis that *ClERF4* is a major gene underlying watermelon rind hardness.

In addition, to explore the *ClERF4* allelic distributions in common watermelon cultivars, 32 cultivars from two panels of protected‐ and open‐field cultivation were analysed using KASP. Interestingly, in general, bb genotypes with lower rind hardness and aa genotypes with higher rind hardness belong to protected and open‐field ecotypes, respectively, which is likely due to artificial selection that occurs in purposeful breeding of rind hardness traits closely associated with *ClERF4* (Table 1). The results undoubtedly suggest that ecotype breeding of watermelon for different cultivation patterns imposes on‐target or off‐target selection of the rind hardness‐related *ClERF4* locus.

**Table 1:** The allelic distribution on elite watermelon cultivars

Varieties	Ecotypes	Genotype
Xinyuchaoxiaolan	Protected‐filed	bb
Caihongyihao	Protected‐filed	bb
Nabite	Protected‐filed	bb
Yuyihuangroujinxin	Protected‐filed	bb
Chunlei	Protected‐filed	bb
Jintaiyang	Protected‐filed	bb
Meidu	Protected‐filed	ab
Lidu	Protected‐filed	ab
Quanyingaoke‐jiale	Protected‐filed	ab
Yuyiguazhibao	Protected‐filed	ab
Lingxian108	Open‐filed	bb
Xinhongbao	Open‐filed	ab
Bingtangtiaozhanzhe	Open‐filed	ab
Chaotianzaobangwang	Open‐filed	ab
Sanzhouban	Open‐filed	ab
Shenmi968	Open‐filed	ab
Meikang9hao	Open‐filed	ab
Quanyingruihu	Open‐filed	ab
Guoyuerhao	Open‐filed	ab
Xiningbahao	Open‐filed	ab
Zhemiliuhao	Open‐filed	ab
Zaojia	Open‐filed	ab
Taiwaiheimeiren	Open‐filed	aa
Hongxiaoyu	Open‐filed	aa
Dileiwang	Open‐filed	aa
Lanhanheimeiren	Open‐filed	aa
Lanhanheimiwang	Open‐filed	aa
Huangjinbaoxigua	Open‐filed	aa
Chaozaowangzi	Open‐filed	aa
Daixin	Open‐filed	aa
Shenkang988	Open‐filed	aa
Xinfeng5hao	Open‐filed	aa

## Discussion

Fruit cracking is episodic in nature, which associated with a number of physiological, biochemical, environmental, cultural, anatomical and genetic factors, causing severe economic losses of flesh fruits (Khadivi‐Khub, 2014). However, precisely quantifying the variations in fruit cracking and phenotyping this phenomenon remains challenging and is becoming a bottleneck for the gene mapping of such agronomically important traits. There were few attempts to phenotype fruit cracking previously, and the prevailing methods include counting the number of cracking fruits and calculating the cracked fruit rate (Capel *et al.*, 2017; Huang *et al.*, 2015). In addition, high levels of water saturation in soil have been employed at the maturation period to evaluate melon cracking capacity (Qi *et al.*, 2015). However, fine mapping of the fruit cracking capacity‐associated genes has not been successful, largely because of the lack of accurate and repeatable indicators to evaluate cracking variability. In the present study, we first used a texture analyser to examine the mechanical properties of watermelon fruits with varied degrees of rind hardness. Interestingly, the indicators CRW, CRT and CRN showed high correlations with rind hardness in the correlation analysis (Tables S1 and S2) and were mapped to the same region by QTL analysis (Fig. 2). Moreover, among the indicators considered, rind hardness is more stable and more reliable. Based our experimental findings, rind hardness is a reliable indicator of cracking resistance capacity that is potentially applicable for gene mapping purposes of watermelon and other fresh fruits.

Combining NGS gene mapping and fine mapping toolkits is efficient and effective ways to identify genes of interest for crop traits (Dou *et al.*, 2018; Li *et al.*, 2018a). Presumably, there is a reasonable chance of overlooking recombinants with statistical methods. Here, we employed haplotype analysis for fine mapping using the VCF data of the target region and found 11 recombinants in 159 F_2_ individuals. Recombinants with chromosome exchange on target regions, such as F_2_ and RIL populations, are often used for fine mapping (Buerstmayr *et al.*, 2018; Dou *et al.*, 2018; Li *et al.*, 2018a; Wang *et al.*, 2018a). However, recombinants in the F_2_ population, whose genome is largely heterozygous, could hardly be used for the fine mapping of the quantitative trait under traditional conditions because the quantitative trait was not as stable as that in F_2_ populations (Fig. 4, Table S8). With the assistance of resequencing and KASP markers, we efficiently identified recombinants in F_2_ and F_3_ populations that contain homozygous domains on the target region. Inspection of the genotypic and phenotypic data from the recombinant F_3_ population zoomed the target region into a 9372 bp fragment (Fig. 5b), where we successfully identified the *ClERF4* gene as a rind hardness regulator (Fig. 5c). Taken together, we concluded that haplotype analysis is an effective strategy to perform fine mapping of traits associated with genes underlying qualitative and near qualitative traits.

Ethylene is one of the most important hormones in plants, and it drives fruit ripening. The ripening process of climacteric fruit is accompanied by a peak in ethylene production and thus results in a dramatic decrease in fruit hardness (Costa *et al.*, 2010). ERFs, signal factors that bridge the internal and external signal and ethylene response, play important roles in stress responses (Liu *et al.*, 2014, 2017, 2018; Zou *et al.*, 2014), growth and development (Yin *et al.*, 2012) and senescence (Tomotsugu *et al.*, 2013). It has been reported that ERFs could be candidate genes for fruit firmness. Tomato firmness QTL Fir^s.p^ 2.1 and Fir^s.p^ 2.5 were identified as harbouring an ERF and three pectin methylesterase (PME) genes (Chapman *et al.*, 2012). An *ETHYLENE‐INSENSITIVE 3‐like* factor transcriptionally regulated the expression of the cell wall hydrolase gene *POLYGALACTURONASE1* (*PG1*) by trans‐activating its promoter in the presence of ethylene and thus controlled the fruit softening process in apple (Tacken *et al.*, 2010). Moreover, a PG‐ethylene‐related gene, *MdACO1*, located on chromosome 10 controlling fruit firmness and fruit softening was found by QTL dynamics on apple (Costa *et al.*, 2010). These findings suggest an important role for ethylene in the reduction of firmness in climacteric fruits. However, the firmness of nonclimacteric fruits such as watermelon does not show such a softening pattern during fruit ripening, and the function of ethylene in rind firmness of nonclimacteric fruits remains unknown. Here, our results demonstrated strong involvement of *ClERF4* in regulating watermelon rind hardness and implied that ethylene is a common regulator of fruit firmness in both climacteric and nonclimacteric fruits.

Accumulating evidence has demonstrated that ERF transcription factors may play roles in lignin biosynthesis and cell wall modification (Lee *et al.*, 2016; Sakamoto *et al.*, 2018; Taylor‐Teeples *et al.*, 2015; Wang *et al.*, 2019; Wessels *et al.*, 2019). By phylogenetic analysis of the ERF superfamily, *ClERF4* was found to be a member of the group IIId ERFs (Figs. S2 and S3). *Arabidopsis* members of this group (*AtERF038*, *AtERF039, AtERF034, AtERF035;* Fig. S3) are possibly involved in modulation of cellulose biosynthesis by transcriptionally regulating PCW‐type CESA genes (Saelim *et al.*, 2019). *Populus ERF139*, another group III ERF, was found to suppress vessel element expansion and stimulate guaiacyl‐type lignin accumulation (Vahala *et al.*, 2013; Wessels *et al.*, 2019). *PpeERF2* was found to bind the promoter region to a cell wall degradation gene (*PpePG1*) and thus to regulate peach fruit ripening (Wang *et al.*, 2019). It was also found that expression of group IIId and IIIe ERF transcription factors in *Arabidopsis* mutants lacking secondary walls results in plants with thickened cell wall characteristics of primary cell walls in the place of secondary cell walls (Sakamoto *et al.*, 2018). We speculate that the function of *ClERF4* in rind hardness variability, and therefore fruit cracking resistance, is possibly related to the regulation of lignin biosynthesis, cell wall modification and/or degradation‐related genes.

In conclusion, this is the first report identifying the causative gene *ClERF4*, from subfamily III, as having a role in fresh fruit rind hardness variability and thus conferring cracking resistance. Clearly, this study provides valuable new insight into the underlying mechanism of rind hardness and fruit cracking resistance. These results will further enable the molecular manipulation of the desirable trait of fruit cracking resistance in fresh fruits such as watermelon via precise targeting of the causative gene *ClERF4,* which is relevant to rind hardness.

## Experimental procedures

### Plant materials and trait measurement

The watermelon accessions P‐a (high rind hardness, cracking tolerant) and P‐b (low rind hardness, cracking sensitive) were selected as parent lines. Their F_1_, F_2_ and F_3_ offspring were obtained by hybridization between P‐a and P‐b and subsequent self‐crossing. Seedlings were grown in a protected greenhouse in Hangzhou, China, in spring of 2018 (parents, F_2_ and germplasm accessions) and 2019 (parents and F_3_). Since both P‐a and P‐b are small‐fruit‐type watermelons, and no significant difference in maturity characters was found among parents and their offspring, Mature fruits were harvested at 30 days after pollination (DAP).

The mechanical properties of the rinds were measured by a Texture Analyzer TA.XT‐21 (Stable Micro Systems Ltd., Godalming, Surrey, UK). For F_2_ and F_3_ populations, only one fruit from each individual was examined; for natural accessions, 3 fruits of each line were employed. The evaluation of rind hardness was examined by a p‐7.5 probe. To minimize the errors caused by the angle of application and spherical stress, three sites on the equatorial zone of each fruit were analysed. The parameters for the measurements were set as follows: the prepressure speed was 1.00 m/s, the test speed was 2.00 mm/s, the posttest speed was 10 mm/s, and the distance was 20 mm. Then, a texture characteristic curve was obtained to quantify the phenotypic trait of rind hardness. The cracking tolerance capacity, including the CRT and CRW, was evaluated using a knife probe (HDP/BS‐B). Only one site on the equatorial zone of each fruit (on the reverse side of the rind hardness measuring point, where the damage of rind should be minimal) was analysed. The parameters for the measurement were set as follows: the prepressure speed was 1.00 m/s, the test speed was 2.00 mm/s, the posttest speed was 10 mm/s, and the distance was 15 mm. Then, we obtained a texture characteristic curve to obtain the phenotypes of the cracking tolerance properties (CRW, CRT). CRW was calculated by the formula of $\text{CRW}=2\times \int_{0}^{7.5}f(x)dx,f\left(x\right)$ was the pressure during the measurement by time, while CRT was the time when the pressure has a sudden decrease (Fig. S4). After the measurements taken with the knife probe HDP/B, the CRN was obtained by measuring the length of the crack. If the crack length was 0 cm, we considered the rind to be none cracked; otherwise, the rind was cracked.

### DNA preparation, quality detection and library construction

DNA was extracted from fresh leaves using Plant DNAzol according to the manufacturer’s recommendations (VWI science). The H‐pool and L‐pool representing high and low rind hardness samples, respectively, were constructed by mixing 20 high‐hardness and 20 low‐hardness F_2_ individuals equally. DNA of 150 bp paired‐end reads was generated with an insert size of approximately 350 bp. Sequencing libraries were generated using the Truseq Nano DNA HT Sample preparation Kit (Illumina), and index codes were added to attribute sequences to each sample according to the manufacturer’s recommendations. Then, the libraries were sequenced using the Illumina HiSeq4000 platform.

The quality of the sequencing data was confirmed using FASTQC (Brown *et al.*, 2017). QC standard pipelines were as follows: reads with a high rate of unidentified nucleotides (≥10%) were removed; reads with a high frequency (>50%) of bases having phred quality less than 5 were removed; reads with more than 10 nt aligned to the adapter were removed; and putative PCR duplicates generated by PCR amplification in the library construction process were removed.

### BSA pipelines

The clean data of four pools were aligned and mapped onto the 97103 reference genome (ftp://cucurbitgenomics.org/pub/cucurbit/genome/watermelon/97103/v2/) by BWA (Burrows‐Wheeler Aligner; Li and Durbin, 2009). Alignment files were converted to BAM files using SAMtools software (Li *et al.*, 2009; settings: –bS –t). In addition, potential PCR duplications were removed using SAMtools command ‘rmdup’. If multiple read pairs have identical external coordinates, only those pairs with the highest mapping quality were retained. SNP calling and InDel filtering were performed using the Unified Genotype function and the Variant Filtration in GATK software (McKenna *et al.*, 2010). ANNOVER software (Wang *et al.*, 2010) was used to annotate SNPs or InDel based on the GFF3 files for the reference genome.

The homozygous SNPs/InDels between two parents were extracted from the VCF files. The read depth information for homozygous SNPs/InDels in the offspring pools was obtained to calculate the SNP/InDel index (Takagi *et al.*, 2013). We used the genotype of one parent as the reference and calculated the statistic read number for this reference parent in the offspring pool. Then, we calculated the ratio of different reads in the total number, which were the SNP/InDel indexes of the base sites. We filtered out those points for which the SNP/InDel indexes in both pools were less than 0.3. Sliding window methods were used to present the SNP/InDel indexes of the whole genome. The average of SNP/InDel index in each window was used as the SNP/InDel index for the given window. The window size of 1 Mb and step size of 10 Kb were employed as default settings. The difference in the SNP/InDel indexes of the two pools was calculated as the delta SNP/InDel indexes. The *G*′ method was conducted by following the instructions of the QTLseqr package in R (Mansfeld and Grumet, 2018).

### Genetic map construction and QTL mapping

PE reads from clean data of two parents and 159 F_2_ individuals were mapped to the reference genome (ftp://cucurbitgenomics.org/pub/cucurbit/genome/watermelon/97103/v2/) using BWA comparison software (parameter: mem ‐t 4 ‐k 32 ‐M ‐R) (Li and Durbin, 2009). Then, SAMtools was used to convert the mapping results into SAM/BAM files (Li *et al.*, 2009), and the comparison rate and coverage were counted with Perl script followed by sorting of the results (parameter: sort) for mutation detection using SAMtools.

Alignment results were filtered to obtain reads that were matched to the unique positions on the genome and were selected for subsequent analysis. For SNP detection and filtering, GATK (‐type UnifiedGenotyper) was used to detect the filtered bam file population SNPs (McKenna *et al.*, 2010). To reduce false‐positive SNPs caused by sequencing errors, the SNP base support numbers from each parent were not <20, and the SNP base support numbers from the offspring were not <2. SNP‐related information, including the heterozygous SNP number, homozygous SNP number and heterozygous SNP ratio, was calculated by Perl script.

Different polymorphic markers from homozygous parents were selected to conduct the SNP genotyping. Then, we filtered the abnormal bases and selected markers to cover <75% of all offsprings. SNPs that significantly deviated from an extreme segregation distortion (*P* < 0.001) were excluded. The high‐quality genetic markers obtained after screening were divided into linkage groups according to the chromosome division method. LepMap3 software was employed to sequence each linkage group using the maximum likelihood method (Rastas, 2017). The Kosambi function was used to calculate the genetic distance between the markers. After removing the markers that could not be confidently interlocked, 5679 bin markers were finally obtained.

The LOD threshold values of each phenotype were determined by PT (permutation test) in MapQTL (https://www.kyazma.nl/index.php/MapQTL/). The CIM algorithm in WinQTL (https://brcwebportal.cos.ncsu.edu/qtlcart/WQTLCart.htm) software was used to locate the QTLs.

### Haplotype analysis

To analyse the haplotype, after the pipeline of SNP callings, we obtained a VCF file containing variation information of parental lines and the F_2_ population. The target region (Cla97Chr10:2534127‐2801352) was withdrawn from the VCF file. The GT of P‐a and P‐b was 1/1 and 0/0 individually and was chosen as the marker for haplotype analysis. We converted the target region results to an Excel file and changed the GT values to ‘a’, ‘b’ and ‘h’. The letters ‘a’, ‘b’ and ‘h’ represented the variants of P‐a, P‐b and heterozygous (h). ‘a’, ‘b’ and ‘h’ were changed to ‘2’, ‘0’ and ‘1’ and labelled with red, blue and yellow colours, respectively.

### Genotyping by KASP

InDel variations on *ClERF4* among the large F_2_ population and germplasm accessions were performed using the KASP platform. The primer combination (Fam, Hex, Common) was used as a marker for genotyping (Table S9). The KASP assay mix was blended with 10 ng/µL FAM, 10 ng/µL HEX and 10 ng/µL R, with a volume ratio of 2:2:5 for the three primers. The KASP reaction was performed in 10.14 µL with 5.0 µL DNA, 5.0 µL KASP master mix and 0.14 µL KASP assay mix. The KASP protocol was utilized as follows: stage 1: preread stage, 30 °C for 1 min; stage 2: hold stage 94°°C for 15 min; stage 3: PCR stage (touchdown), 94 °C for 20 s, 61°°C for 1 min (decrease pf −0.6 °C), recycling for 9 times (a total of 10 cycles), achieving a final annealing temperature of 55°°C; stage 4: PCR stage, 94 °C for 20 s, 61°°C for 1 min, recycling for 25 times; and stage 5: postread stage, 30°°C for 1 min. After the amplification, an ABI PRISM 7900HT (Applied Biosystems) was used to detect the fluorescence signal and validate the classification. If the genotyping was not sufficient, the protocol was expanded, stages 4 and 5 were continued for 3 more cycles, and the results were checked to confirm completion; then, the experimental results were derived from the machine.

### Identification of the ERF gene family in watermelon and polygenetic analysis

The hidden Markov model (HMM) for ERF (PF00847) was obtained from the PFAM database (http://pfam.xfam.org/), and the model was used to query for the ERF gene family of watermelon. The *Arabidopsis* ERF gene protein sequence was obtained from the TAIR database (https://www.arabidopsis.org/), and the subfamily information was acquired from a previous study (Nakano *et al.*, 2006). A neighbouring joint tree was constructed using MEGA7.0 based on the full‐length protein alignment.

## Conflict of Interest

The authors declare that there is no conflict of interest.

## Author contributions

MZ and ZH conceived and designed the experiments. NL, JH, SC, XQ, GKM, AA and AM developed the F2 and F3 populations and measured the phenotypes. NL, YL, YM and KJ performed DNA extraction and genotyping. NL and ZH performed QTL‐seq and genetic map analysis as well as prepared the first draft, and NL, ZH and MZ contributed to the final editing of the manuscript. All authors read and approved the final manuscript.

## Supporting information


**Figure S1** The genetic map of watermelon based on re‐sequencing of 159 individuals from F_2_ populations

**Figure S2** The phylogenetic tree of EFRs from watermelon and *Arabidopsis.*

**Figure S3** The phylogenetic relationships and protein structures of the group III ERFs from different species.

**Figure S4** The texture characteristic curves for the measurement of rind hardness (RH) and the cracking‐tolerance properties (CRW, CRT).

**Table S1** The relationship among rind mechanical properties indicators in F_2_ population
**Table S2** The relationship among rind mechanical properties indicators in nature genetic accessions
**Table S3** The sequence data summary for genetic map
**Table S4** The QTL results of rind mechanical properties
**Table S5** The sequence data summary of BSA project
**Table S6** The QTL results of rind hardness on 95% confidence intervals
**Table S7** The QTL results of rind hardness on 99% confidence intervals
**Table S8** The QTL results of rind hardness on 99% confidence intervals analysed by QTL‐seqr
**Table S9** Details of newly developed SNP markers
**Table S10** The rind hardness and genotyping of F_2_ individuals
**Table S11** The rind hardness and genotyping on 104 germplasm accessions
