# Supplementary figures and images for: Ethylene‐responsive factor 4 is associated with the desirable rind hardness trait conferring cracking resistance in fresh fruits of watermelon
Source: Plant Biotechnol J. 2019 Nov 6;18(4):1066–77. doi: 10.1111/pbi.13276 (PMC7061880; doi:10.1111/pbi.13276)

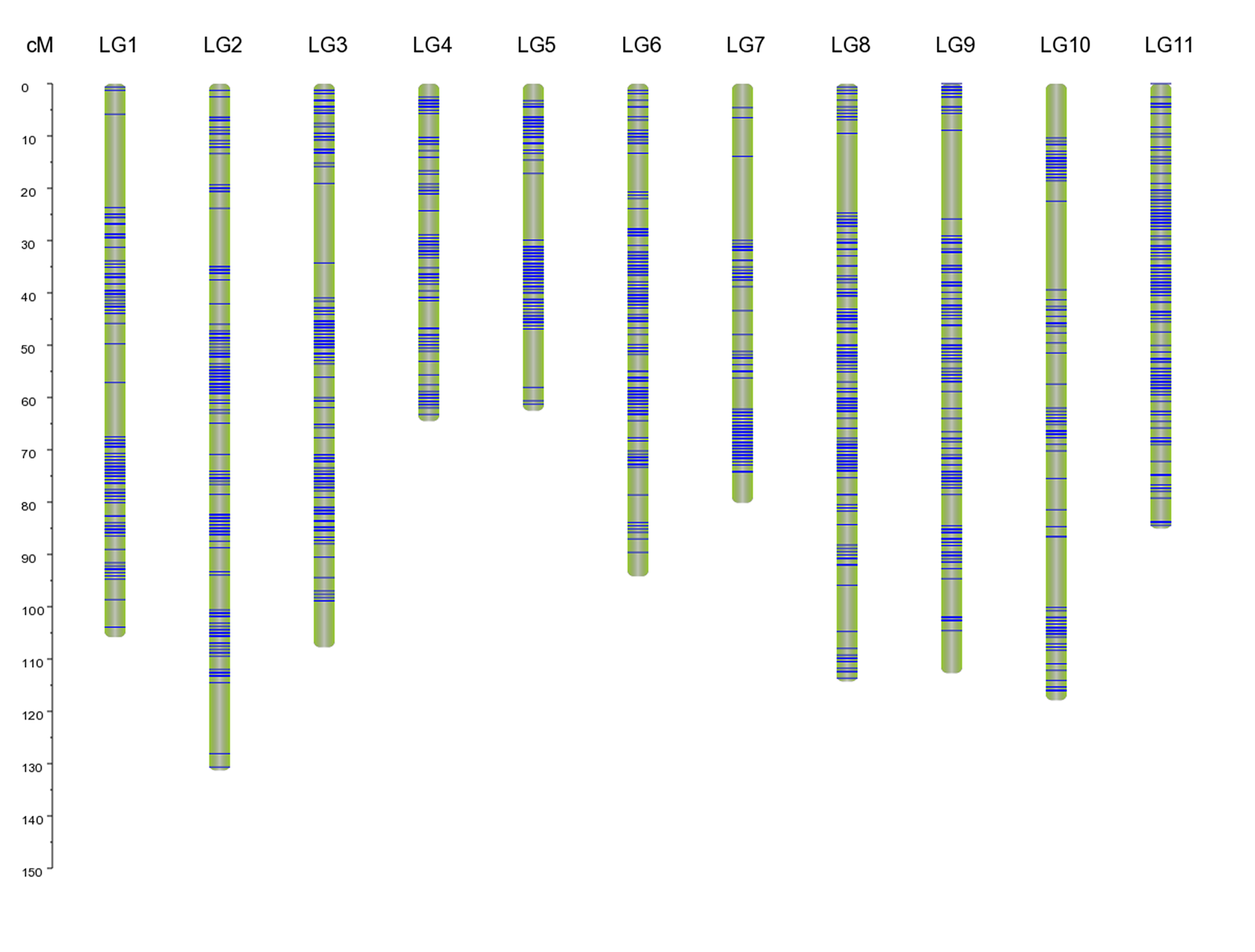

Supplement: Supplementary file 1 — Figure S1 The genetic map of watermelon based on re‐sequencing of 159 individuals from F2 populations [file PBI-18-1066-s004.png]

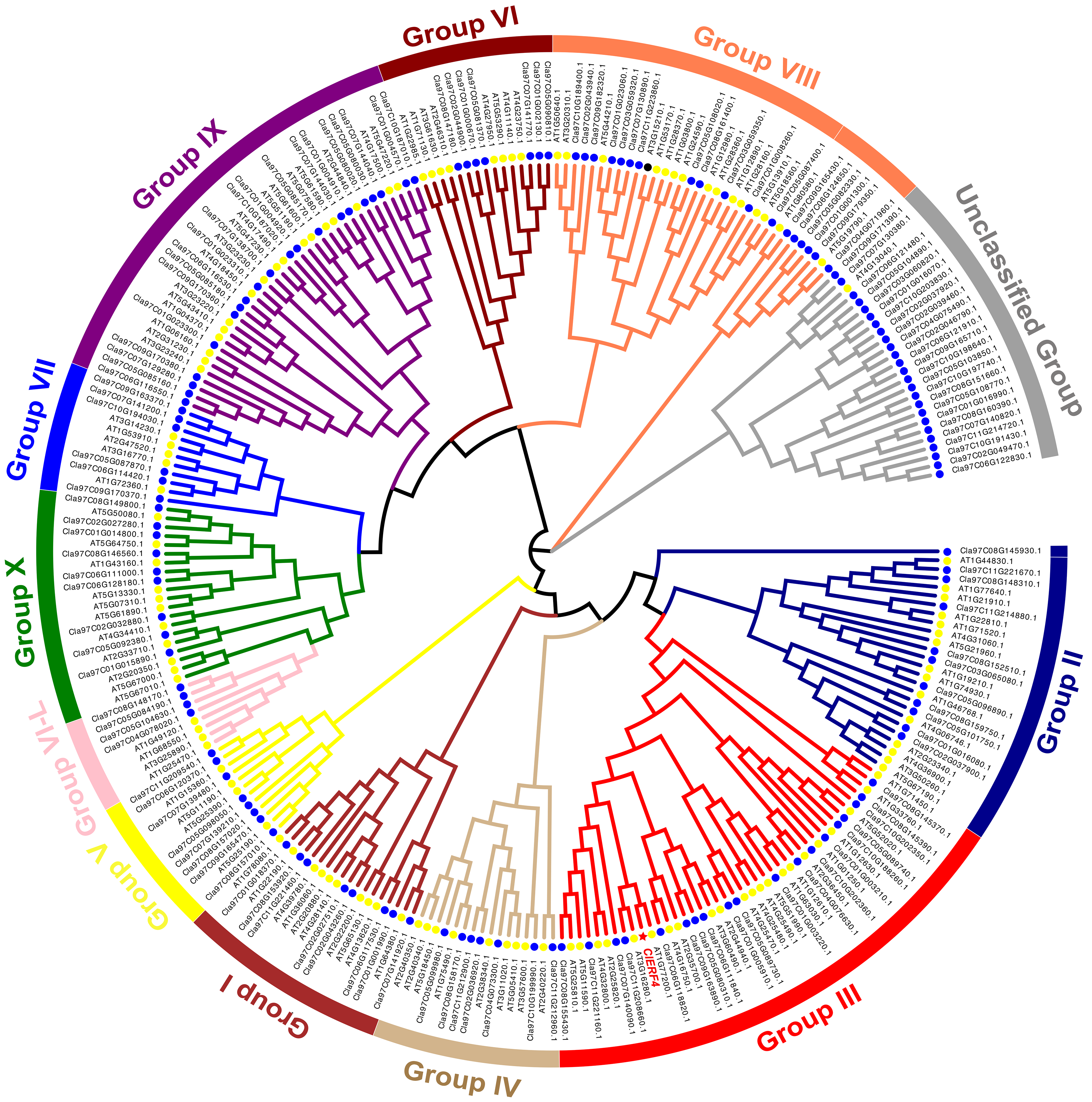

Supplement: Supplementary file 2 — Figure S2 The phylogenetic tree of EFRs from watermelon and Arabidopsis. [file PBI-18-1066-s003.png]

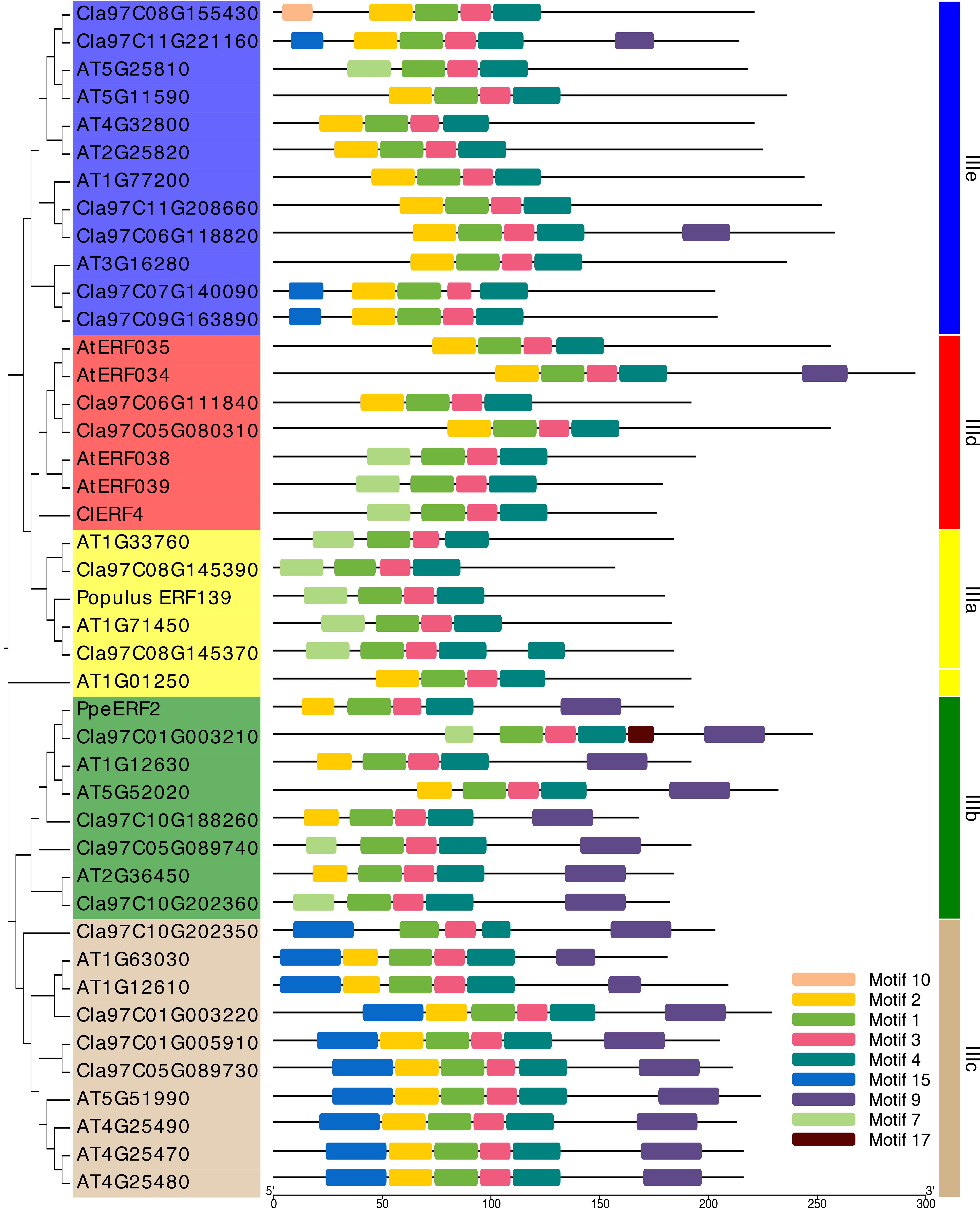

Supplement: Supplementary file 3 — Figure S3 The phylogenetic relationships and protein structures of the group III ERFs from different species. [file PBI-18-1066-s005.png]

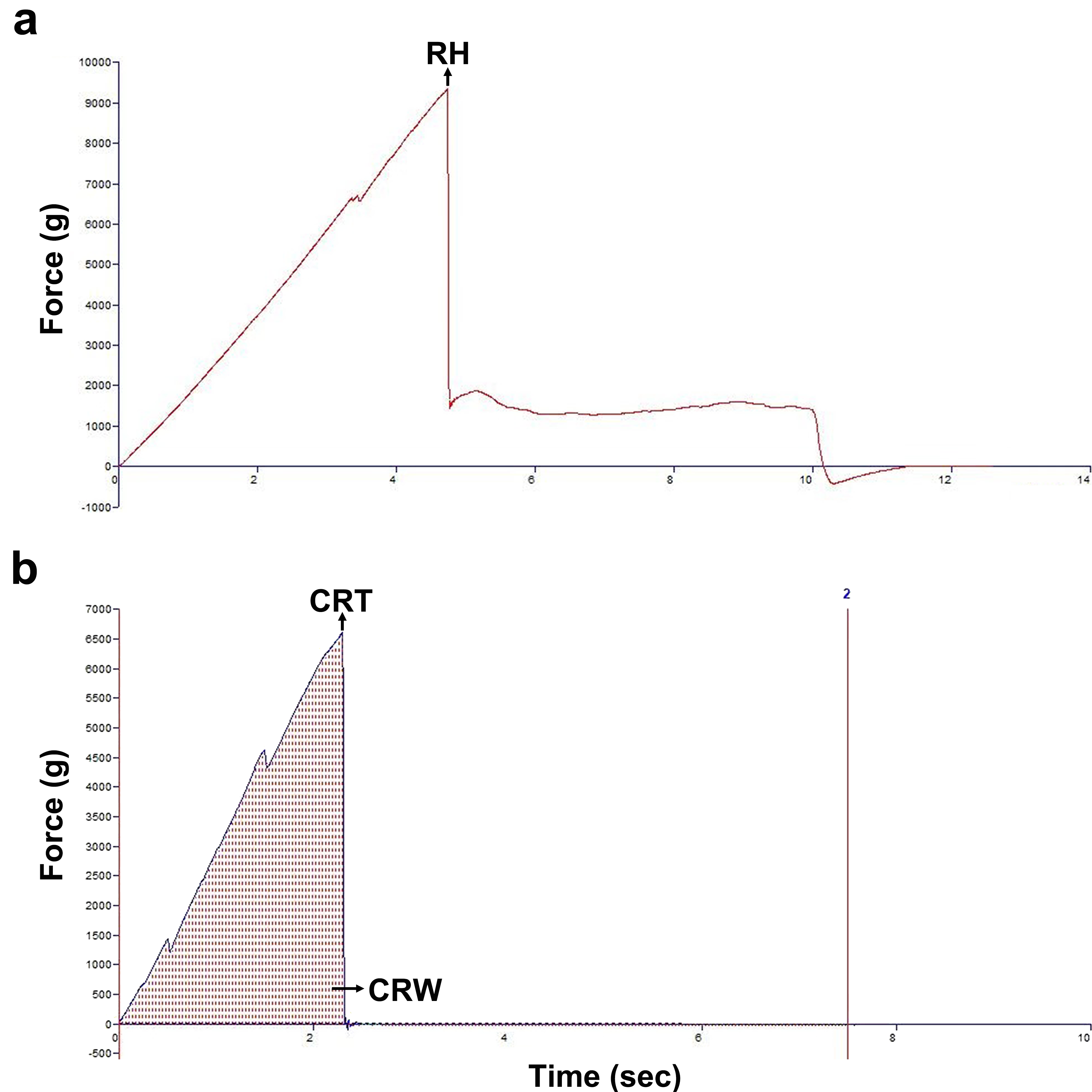

Supplement: Supplementary file 4 — Figure S4 The texture characteristic curves for the measurement of rind hardness (RH) and the cracking‐tolerance properties (CRW, CRT). [file PBI-18-1066-s002.png]
